# Federated Learning-Based Detection of Invasive Carcinoma of No Special Type with Histopathological Images

**DOI:** 10.3390/diagnostics12071669

**Published:** 2022-07-09

**Authors:** Bless Lord Y. Agbley, Jianping Li, Md Altab Hossin, Grace Ugochi Nneji, Jehoiada Jackson, Happy Nkanta Monday, Edidiong Christopher James

**Affiliations:** 1 School of Computer Science and Engineering, University of Electronic Science and Technology of China, Chengdu 611731, China; agbleybless@outlook.com (B.L.Y.A.); mh.nkanta@std.uestc.edu.cn (H.N.M.); 2 School of Innovation and Entrepreneurship, Chengdu University, Chengdu 610106, China; altabbd@163.com; 3 School of Information and Software Engineering, University of Electronic Science and Technology of China, Chengdu 611731, China; ugochinneji@std.uestc.edu.cn (G.U.N.); kofijackson@uestc.edu.cn (J.J.); edianajames@yahoo.com (E.C.J.)

**Keywords:** breast cancer, deep learning, federated learning, invasive carcinoma of no special type, whole slide images, histopathological image analysis

## Abstract

Invasive carcinoma of no special type (IC-NST) is known to be one of the most prevalent kinds of breast cancer, hence the growing research interest in studying automated systems that can detect the presence of breast tumors and appropriately classify them into subtypes. Machine learning (ML) and, more specifically, deep learning (DL) techniques have been used to approach this problem. However, such techniques usually require massive amounts of data to obtain competitive results. This requirement makes their application in specific areas such as health problematic as privacy concerns regarding the release of patients’ data publicly result in a limited number of publicly available datasets for the research community. This paper proposes an approach that leverages federated learning (FL) to securely train mathematical models over multiple clients with local IC-NST images partitioned from the breast histopathology image (BHI) dataset to obtain a global model. First, we used residual neural networks for automatic feature extraction. Then, we proposed a second network consisting of Gabor kernels to extract another set of features from the IC-NST dataset. After that, we performed a late fusion of the two sets of features and passed the output through a custom classifier. Experiments were conducted for the federated learning (FL) and centralized learning (CL) scenarios, and the results were compared. Competitive results were obtained, indicating the positive prospects of adopting FL for IC-NST detection. Additionally, fusing the Gabor features with the residual neural network features resulted in the best performance in terms of accuracy, F1 score, and area under the receiver operation curve (AUC-ROC). The models show good generalization by performing well on another domain dataset, the breast cancer histopathological (BreakHis) image dataset. Our method also outperformed other methods from the literature.

## 1. Introduction

Breast cancers are among many diseases the research community is working hard to detect with the aid of automated systems [1,2,3,4]. While tumors can be benign or malignant, the former is not considered cancerous as their cells are more regular, develop more slowly, and are not invasive of tissues around them. Malignant tumors, on the other hand, are cancerous tumors. The term “breast cancer” is used to refer to malignant tumors originating from the lobules or ducts of the breast or, in rare cases, the stromal tissue (the connective fatty and fibrous breast tissues), which are then further propagated to other parts of the body by the lymphatic system [5]. They are caused by genetic abnormalities where about 5% to 10% of the cases are due to inheritance from parents. About 90% of the hereditary cases are a result of mutations in the breast cancer genes BRCA1 and BRCA2 [6,7,8,9]. Breast cancer makes up 30% of all new cancer cases diagnosed in women. The American Cancer Society estimates 287,850 new invasive cancer cases in women in 2022, and 43,250 individuals will die of the disease. Additionally, an estimate of 2710 men is expected to be diagnosed with invasive breast cancer in 2022. Out of this number, about 530 are likely to die from it [10].

Invasive carcinoma of no special type (IC-NST), previously referred to as invasive ductal carcinoma (IDC) or breast cancer not otherwise specified (NOS) [11], is known to be a very prevalent kind of breast cancer, making up about 80% of all breast cancers according to the American Cancer Society.

The procedure for diagnosing breast cancer involves a combination of different tests, including physically examining the breast, mammograms, and biopsy. Other examinations with ultrasound and breast magnetic resonance imaging (MRI) may also be considered [12]. The study carried out by Collins et al. indicated that core needle biopsy provides high rates for diagnosing IC-NST in over 90% of the cases, and it is a primary technique for evaluating histopathological features among pathologists [13]. The process, however, involves manual feature engineering by expert pathologists who must carefully observe and examine the glass slide of biopsy specimens. Capturing these specimens into images makes them available for use in computer-aided detection/diagnosis [14]. Approaches such as machine learning (ML) and, more specifically, deep learning (DL), have been explored in recent years due to their successes in aiding in the prognosis and diagnosis of other medical conditions [15,16,17,18,19,20,21,22]. These techniques provide mathematical models for automating the detection process. However, to effectively take good advantage of the ability of DL models to eliminate manual feature engineering, the methods tend to require large amounts of data. The regulations governing the release of patient data are, however, stringent, resulting in a small amount of publicly available data.

To address this privacy concern and encourage collaborative learning, we employ federated learning in this study. This paper contributes the following to help in the automated detection of IC-NST:

- We proposed a multimodal network by introducing two input modalities obtained separately by extracting histopathology image features with GaborNet and pre-trained ResNet.
- We formulated the problem of automatically diagnosing IC-NST as a federated learning challenge to leverage its privacy preservation capability.
- We conducted experiments to evaluate our approach and compared the results against some techniques in the literature in terms of accuracy, F1-score, and balance accuracy metrics.
- We also assessed how well our models generalized by evaluating them on another domain dataset from a different repository.
- Visualizations of the layers of both ResNet and GarborNet were provided to give more insight into the models and enhance their explainability.

We organized the subsequent sections in the following manner. Section 2 presents literature related to IC-NST and motivation for the study. In Section 3, we present our methodology. We implemented our approach, presented our experimental results, and compared them with other methods proposed in the literature in Section 4. Finally, we concluded in Section 5 and presented some future research directions.

## 2. Related Works

IC-NST detection has been a very important topic for researchers applying ML and DL in addressing breast cancer issues. Romano and Hernandez [23] performed a study on IC-NST in which they trained an enhanced convolutional neural network (CNN) model and analyzed the results of their model with a patch-based IC-NST classification problem. Their study achieved an F-score and balanced accuracy of 85.28% and 85.41%, respectively. Brancati et al. [24] explored some techniques to automatically analyze hematoxylin and eosin (HE)-stained histopathological images of breast cancer and lymphoma. They also proposed their approach for two use cases: the detection of IC-NST in breast histopathological images and the classification of lymphoma subtypes. They reported having improvements of 5.06% in the F-score and 1.09% in the accuracy measure over previous methods. Liu et al.  [25] proposed a new index of the energy to Shannon entropy ratio (ESER), which they used for classifying the tissues. They utilized principal component analysis (PCA) together with an ML technique to autonomously classify THz signals from IC-NST samples and had AUCs greater than 0.89, 92.85% precision, 89.66% sensitivity, and 96.67% specificity. Chapala et al. [26] proposed the use of the ResNet50 framework to automatically detect IC-NST from breast histopathology images (BHI). Their model achieved an accuracy of 91%. Celik et al. [27] conducted a study involving the automatic detection of IC-NST using a deep transfer learning technique. They used some pre-trained models of DL, including ResNet50 and DenseNet161, on BHI for their study. They obtained an F-score of 92.38% and a balanced accuracy value of 91.57% on the DenseNet161 model. They also obtained an F-score of 94.11% and a balanced accuracy value of 90.96% on the ResNet-50 model. Chand et al. [28] performed a more inclusive study on the various methods used over the recent years for IC-NST detection.

Transfer learning (TL) is a popular technique used to improve the performance of models for which there are inadequate training samples. They are also very convenient for reducing the training time by basing on the pre-trained weight of earlier layers in the network to fine-tune new models. Popular TL architectures are trained with the ImageNet dataset. For a medical use case such as the task of IC-NST detection, ImageNet-based transfer learning methods become out of the domain. Hence, Laith et al. [29] proposed a novel TL technique for medical imaging tasks with limited labeled datasets. They take advantage of the unlabeled medical data available to train models that learn domain-specific features, and the learned weights are used to fine-tune models for which there are limited datasets. The authors also proposed a new deep convolutional neural network (DCNN) to exploit recent advances in the area. To evaluate their proposed TL and DCNN models, they performed separate experiments involving automated skin cancer and breast carcinoma classification. Among the many promising results achieved by their method, their model obtained an increment from an 86.0% F1-score when trained without any form of TL to 96.25% with TL, as well as an even higher F1-score of 99.25% with the double-TL technique on a two-class (normal and diabetic foot ulcer) skin classification task. In a similar paper, the authors [30] proposed a hybrid DCNN to classify HE-stained breast biopsy images into four targets (invasive carcinoma, in situ carcinoma, benign tumor, and normal tissue). They empirically showed that domain-based TL does well at optimizing the performance of the models. Augmenting the training instances reduced overfitting by providing more instance variations for the model to explore for generalization.

Despite the promising results of all the methods proposed in the literature involving DL and TL techniques, privacy concerns regarding health data add more limitations to the availability of publicly accessible data. Institutions are usually obliged to run models on their localized datasets to keep their patients’ data private. Motivated by the successful use of FL by Google to achieve a high performing model for predicting words on their Gboard [31,32], some recent works in computerized medical image analysis [19] have adopted FL to help protect patients’ privacy. This work also uses FL to bridge the IC-NST data availability gap and to encourage collaboration between different institutions without sharing their patients’ data.

## 3. Proposed Approach

Data scarcity affects the generalization of deep learning models as there are not enough instance variations to represent the distribution under study adequately. This is a big challenge; hence, institutions resort to collaborating by donating their data to a common repository [33] for centralized learning (CL). While this helps in addressing the challenge of availability of big data to improve model generalization, it also introduces the challenge of data privacy [34]. This is a significant concern in health, adding to the already existing data limiting factors such as the lack of experts to annotate and digitize traditionally acquired data.

**Figure 1:**
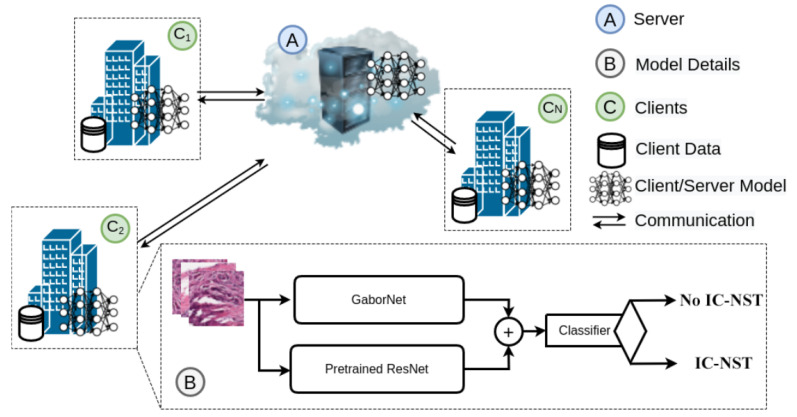
Architecture of the proposed approach with GaborNet and ResNet models on a federated setting.

We proposed federated learning-based IC-NST detection in which the clients utilize pre-trained residual models and the Gabor network to extract features from their private IC-NST datasets. The architectural diagram of the proposed model is represented in Figure 1. It consists of the global FL model on the sever (A), the client model architectures residing on each client node (B), and the private datasets for each client, also residing on the client node. More details on our approach are provided in the following sub-sections.

### 3.1. Federated Learning (FL)

FL eliminates the need for one common data bank for access by the participating institutions. Their data remain with them while they contribute training parameters to help improve each other’s local models. We present our proposed FL approach in Algorithm 1. Using federated averaging [31], the parameters of each local client model are averaged on a central server. These averaged parameters are then used to update the participating clients for a number of rounds. For each round of server update, each of the clients trains its local model for several epochs. Given *N* number of clients, each client has its own local IC-NST patch dataset, $X_{i}$ and, labels, $Y_{i}$, where *i* specifies the client. The set of all the client data are $\left\{X_{1},\ldots ,X_{N}\right\}$ with labels, $\left\{Y_{1},\ldots ,Y_{N}\right\}$. A global model *M* is agreed upon by all the clients, and a clone $m_{i}$ is made by each of the clients and initialized with the parameters of *M*. The entire local model list is thus $\left\{m_{i},\ldots ,m_{N}\right\}$ parameterized with $w_{i}=W$, where $w_{i}$ is the local parameters whiles *W* represents the global parameters.

The server aggregation phase is carried out after each round of local training. The local parameters $\left\{w_{i},\ldots ,w_{N}\right\}$ are transmitted to the server for aggregation using the federated averaging (FedAvg) algorithm to compute the average of all the local parameters, which becomes the new global parameter, *W*, as shown in Equation (1) below:

(1) $$\underset{w}{\mathrm{minimize}}\;F\left(w\right):=\frac{1}{N}\sum_{i=1}^{N}F_{i}\left(w\right),$$

where $F_{i}\left(w\right)$ represents the risk function for client *i*. The risk function is further presented in Equation (2) below:

(2) $$F_{i}\left(w\right)=E_{\left(x_{i},y_{i}\right)∼D_{i}}\left[L(m_{w}\left(x_{i}\right),y_{i})\right],$$

where $D_{i}$ represents the distribution of each client data, L represents the loss function, $m_{w}$ represents the local model, and $m_{w}\left(x_{i}\right)$ represents the model prediction for a data instance $x_{i}$. Approximating the risk with an empirical function, we have Equation (3):

(3) $$F_{i}\left(w\right)\approx \frac{1}{n_{i}}\sum_{i=1}^{n_{i}}\left[L(m_{w}\left(x_{i}\right),y_{i})\right]$$

We used the cross entropy loss function for the optimization of our models. For balanced classes, the loss is formulated as follows:

(4) $$L\left(m_{w}\left(x_{i}\right),y_{i}\right)=-\log\left(\frac{\exp(x[y])}{\sum_{j}\exp\left(x\left[j\right]\right)}\right)$$

(5) $$L\left(m_{w}\left(x_{i}\right),y_{i}\right)=-x\left[y_{i}\right]+\log\left(\sum_{j}\exp\left(x\left[j\right]\right)\right)$$

**Algorithm 1:** Proposed FL-Based Approach.

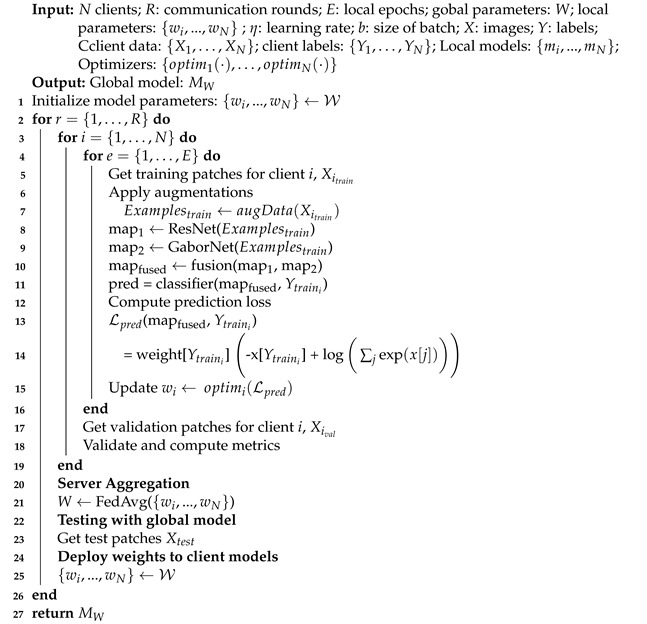



### 3.2. FL Model Architecture

Taking advantage of TL for fine-tuning models trained on smaller datasets [35,36,37,38], this study utilizes residual network models pre-trained with the ImageNet dataset for automatic feature extraction from the histopathology patches to produce one feature modality. A second feature modality is obtained using a Gabor network.

ResNet: ResNet18 and ResNet50 are two of the deep learning networks proposed by He et al. [39], in which they showed that residual learning by referencing the layer’s input enables the construction of deeper networks without increasing the error. Similar to the original ResNet18 and 50 networks, our implementation consists of a convolutional layer with 7×7 filters, an output channel of 64, a stride of 2, and padding of 3×3. Following the convolution layer is a 3×3 max-pooling with a stride of 2. Four sequential layers consisting of blocks of convolution, batch normalization, and ReLU layers are then added. The major difference between the ResNet18 and 50 networks is the number of blocks used to compose each layer. While ResNet18 consists of 2 blocks per layer, ResNet50 contains 3, 4, 6, and 3 blocks, respectively, for each layer. Average pooling is added after the last layer of both networks. We modified the linear layer in the fully connected block to output a feature size of 448.

GaborNet: Gabor filters are linear band pass filters that analyze frequencies in localized regions for directions defined by the kernel [40,41]. Given a kernel size $\left(p,q\right)$, phase offset, ψ, spatial aspect ration, γ, and standard deviation, σ, a complex Gabor kernel is obtained using the function $g\left(\cdot \right)$ in Equation (6). The real and imaginary components are Equations (7) and (8), respectively.

(6) $$g\left(\cdot \right)=\exp\left(-\frac{{p^{\prime }}^{2}+\gamma^{2}{q^{\prime }}^{2}}{2\sigma^{2}}\right)\exp\left(i\left(2\pi \frac{p^{\prime }}{\lambda }+\psi \right)\right)$$

(7) $$g\left(\cdot \right)=\exp\left(-\frac{{p^{\prime }}^{2}+\gamma^{2}{q^{\prime }}^{2}}{2\sigma^{2}}\right)\cos\left(2\pi \frac{p^{\prime }}{\lambda }+\psi \right)$$

(8) $$g\left(\cdot \right)=\exp\left(-\frac{{p^{\prime }}^{2}+\gamma^{2}{q^{\prime }}^{2}}{2\sigma^{2}}\right)\sin\left(2\pi \frac{p^{\prime }}{\lambda }+\psi \right)$$

Here, $p^{\prime }=p\cos\theta +q\sin\theta$ and $q^{\prime }=-p\sin\theta +q\cos\theta$. A total of 96 kernels were defined by varying the function parameters to produce different frequencies at different directions. We used the kernels along with 2D convolution to extract features from the images. Another convolutional layer with a 3×3 kernel size and an output size of 384 are added. We also added a linear layer consisting of 64 output size.

Classifier: The features obtained from the ResNet $\left(map_{1}\right)$ and the features extracted with the GaborNet $\left(map_{2}\right)$ are fused [19,42] and fed into a classifier for prediction. The classifier consists of a linear layer with a 256 output size, a ReLU activation layer, a batch normalization layer, a dropout of 0.5, and a final linear layer with an output of 2 for the two classes.

### 3.3. Centralized Learning (CL)

Since the major practice over these past years is to have the training data centralized in one device or server, we considered CL our benchmark technique for comparison with the proposed FL approach. The CL technique involves running the same deep learning networks described in Section 3.2 for the FL approach in a non-distributed fashion. Hence, the datasets are kept at one location, and the various model variations are evaluated on them. Those models specific to the CL are named CL+ResNet18, CL+ResNet18+Gabor, CL+ResNet50 and CL+ResNet50+Gabor. CL in the naming refers to the centralized learning mode, while ResNet18, ResNet50, and GaborNet refer to the feature extraction techniques. The “+” symbol implies the combination of different techniques.

### 3.4. Datasets

Deep learning relies heavily on datasets to automatically extract features that uniquely characterize the various target classes. We used two histopathological datasets from different repositories for our study. These datasets are described in the following sub-sections.

#### 3.4.1. Breast Histopathology Image (BHI) Dataset

**Figure 2:**
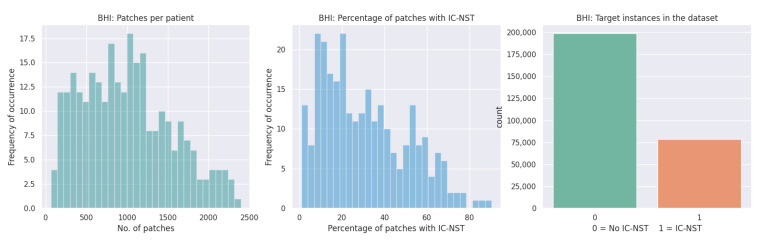
Patches available in the BHI dataset.

**Figure 3:**
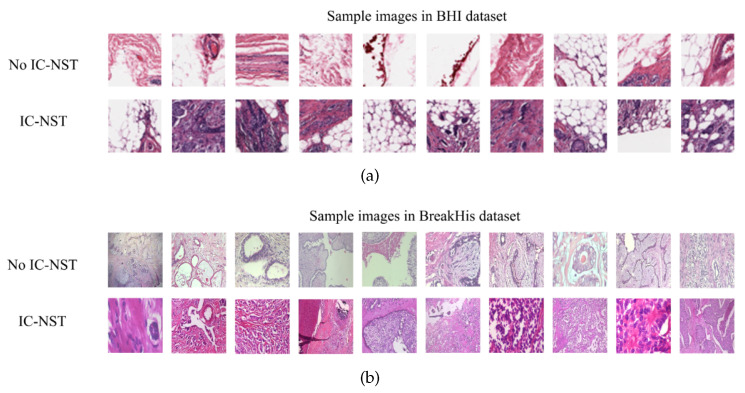
Histopathology image samples in the (**a**) BHI and (**b**) BreakHis datasets.

We utilized the Breast Histopathology Image (BHI) dataset [41,43,44] for training. The original dataset contains whole slide images (WSI) taken at 40× magnifying factor. In total, 277,524 patches of size 50×50 were obtained from the whole slide images, of which 198,738 patches are for IC-NST-negative and 78,786 are for IC-NST-positive cases, as shown in Figure 2. Samples of the two classes in the BHI dataset are shown in Figure 3a.

**Figure 4:**
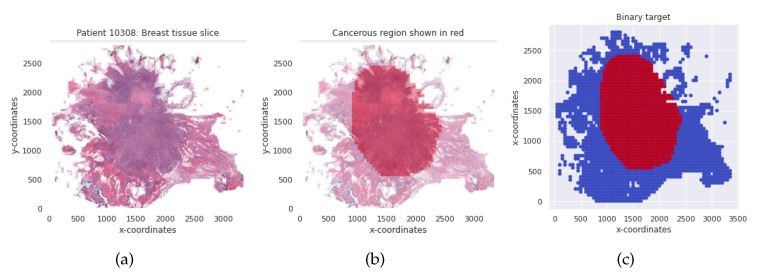
(**a**) Breast tissue slice for an IC-NST subject. (**b**) The red colored mask shows the cancerous region. (**c**) Binary target per tissue slice for the IC-NST subject.

Considering a subject from the BHI dataset, for instance, the corresponding patches are aligned to enable us to visualize the entire tissue without a mask of the malignant tumor region as shown in Figure 4a. The region indicated by the deep red mask is the cancerous region, shown in Figure 4b. The tissue slice with the binary positive and negative target patches are also shown in Figure 4c. The pre-processing included augmenting the training and validation patch instances by randomly flipping the images horizontally and vertically and then applying a z-score normalization.

We identified 279 unique patients in the BHI dataset; hence, we obtained an identically independent distribution (IID) by allocating 93 patients randomly to each client. All the patches of the patients assigned to the clients were used to train their private models.

#### 3.4.2. Breast Cancer Histopathological (BreakHis) Image Dataset

Samples obtained through surgical (open) biopsy (SOB) were prepared into histopathological images to create the Breast Cancer Histopathological Image dataset by staining with hematoxylin and eosin (HE) [45,46,47]. The microscopic breast tumor images were taken at four different magnifying factors: 40×, 100×, 200×, and 400×. A total of 9109 images were present, of which 2480 belonged to the benign class, whiles 5429 belonged to the malignant class. The images were 3-channel RGB images of size 700×460. Based on the appearance of the cells, the images can be categorized into the benign (adenosis, fibroadenoma, phyllodes, and tubular adenoma) class and the four invasive cancer subtypes (ductal carcinoma, lobular carcinoma, mucinous carcinoma, and papillary carcinoma). Samples in this dataset are graphically presented in Figure 3b.

In order to handle the difference in the sizes between the BHI and BreakHis datasets, we cropped 50×50 patches from the center of all the images considered for evaluation from the BreakHis dataset. Cropping from the center retains information about the context without losing the target class.

#### 3.4.3. Handling Imbalance Data Label for Loss Computation

There is an imbalance in the number of instances in the target classes. Imbalanced classes tend to learn the class with larger training instances more effectively than the class with the smaller instances, thereby affecting the overall model performance. To overcome this issue, we computed the weights, given each class, *j* in {1,…,J}, for each client as follows:

(9) $$\mu_{i,j}=\frac{|X_{i}|}{J\times |Y_{i,j}|}$$

where $|X_{i}|$ is the number of instances for client *i*, and $|Y_{i,j}|$ is the total number of instances in class *j* for client *i*. The loss function in Equation (5) is modified to factor in the weight of each class as follows:

(10) $$L\left(m_{w}\left(x_{i}\right),y_{i}\right)=\mu_{i,j}\left(-x\left[y_{i}\right]+\log\left(\sum_{j}\exp\left(x\left[j\right]\right)\right)\right)$$

## 4. Experiments

### 4.1. Experiment

Different sets of experiments were performed on both the CL and FL setups. Specifically, we ran experiments using only ResNet18 and ResNet50 for feature extractions and also combined the GaborNet for each of the cases. We performed training, validation, and testing using the BHI dataset. Exploring the dataset, 279 unique patients were identified, and hence we allocated 93 patients to each of the clients. This ensured that the patches owned by each of the clients belong to a patient assigned to them. Thus, every patch located on each client node was private to that client. The patches at each client node were then split into train, dev and test sets of 70%, 15% and 15% respectively. The train and dev sets, shown in Table 1, were kept private to each client. The test sets at each client node are combined to produce one set for evaluating the global model. Whiles each client validated their respective models on their dev-set, the combined test-set was used in evaluating the global model. The data augmentation techniques used for both the training and development sets at the client level included random horizontal and vertical flips and z-score normalization. During training, local parameters were sent to the server for averaging after every 2 epochs for a total of 30 rounds. Due to the imbalanced nature of the dataset, we computed the class weights for each client according to Equation (9) using the weighted cross-entropy loss function as our criterion. The stochastic gradient descent (SGD) with varying learning rates was used. We scheduled the learning rate using Equation (11):

(11) $$lr_{next}=lr_{1}\times 0.085^{e+1},$$

where the initial learning rate was $lr_{1}$ = 1.0, and $e\in N^{+}$. A batch size of 512 was used for all the experiments.

**Figure 5:**
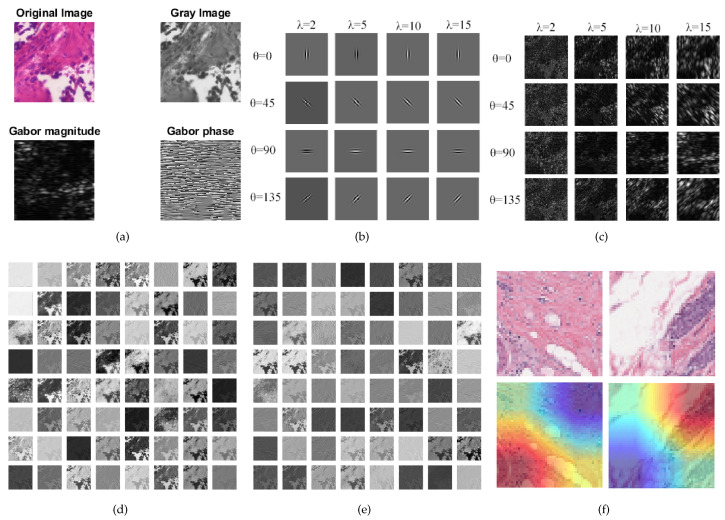
Visualizations: (**a**) Original histopathology image, gray-scale image and the Gabor magnitude and phase for wavelength λ=10 and orientation θ=90. (**b**) Sample Gabor filter bank with different λ and θ values. (**c**) Features extracted with the filter bank. (**d**) Features learned by the first CNN layer of ResNet50. (**e**) Features learned by the second CNN layer of ResNet50. (**f**) Using Grad-CAM to visualize some images.

We provide a visualization of the features extracted by the Global FL+ResNet50+Gabor model in Figure 5. In Figure 5a, we show a sample of an original malignant image and its corresponding gray image. We also show the magnitude and Gabor phase obtained after applying a filter with a wavelength of 10 at an orientation of 90 degrees to the image. In Figure 5b, we show a grid of different filters (filter bank) used to extract different orientations of frequency-based features from the sample image. The extracted features are shown in Figure 5c. Next, Figure 5d,e show the features extracted by the first and second layers of the model. Finally, we visualized the model using Grad-CAM to provide insight on the model’s localization for its predictions [48]. We used PyTorch and an NVIDIA GeForce RTX 2080 Ti accelerator with a memory of 11 Gb for the experiments.

### 4.2. Metrics, Results, and Discussion

For each experiment, we calculated the true positive predictions (TP), the false positive predictions (FP), the true negative predictions (TN), and the false negative predictions (FN). These were further used to compute commonly used metrics for medical data classification tasks [24,27,49,50] and the performance shown in Table 2 and Table 3. Columns $C_{1}$, $C_{2}$, and $C_{3}$ in the tables refer to client 1, client 2, and client 3, respectively. *G* refers to the global model (obtained by the FL) or the centralized model.

Specificity is a measure of the proportion of negatives the model classified correctly. Specificity is computed with

(12) $$Specificity=\frac{TN}{TN+FP}.$$

From the result, the best specificity performance was attained by the federated model using ResNet18 and GaborNet features. We noticed that the results obtained by the FL models outperformed the CL models with respect to the specificity metrics. Additionally, fusing the features from the GaborNet and the ResNets tended to further improve the specificity.

Recall gives the proportion of positives correctly classified by the model and is obtained with the equation

(13) $$Recall=\frac{TP}{TP+FN}\times 100.$$

From Table 2, the best recall performance of 82.15% was attained when we performed centralized learning with both ResNet18 and GaborNet features. Likewise, the subsequent best recall of 79.90% was obtained by training the CL model with both ResNet50 and GaborNet feature extractors.

Precision reflects the accuracy of the positive predictions. It is obtained by dividing the TP by the total positives, as shown in Equation (14).

(14) $$Precision=\frac{TP}{TP+FP}$$

The best precision, 80.05%, was obtained by using both ResNet18 features and the GaborNet features on FL. Using ResNet50 and GaborNet features on FL resulted in the next best result of 79.60%.

Accuracy measures the percentage of correctly classified cases, including both the positive and negative classes. The computation is shown in the following equation:

(15) $$Accuracy=\frac{TP+TN}{TP+TN+FP+FN}\times 100.$$

From Table 3, the accuracy obtained by training ResNet18 in a centralized fashion with and without the Gabor features are at par with each other with a difference of only 0.02%. Combining GaborNet and ResNet18 feature extractors in a FL fashion produced an improvement of 0.35% over using only ResNet18 for FL. For ResNet50, the centralized model without the Gabor features performed better than its Gabor feature combination.

The $F_{1}$ score calculates the harmonic mean of the precision and sensitivity as formulated in Equation (16).

(16) $$F_{1}=2\times \frac{precision\times sensitivity}{precision+sensitivity}$$

The best $F_{1}$ score (86.49%) was obtained by the FL model trained on both ResNet50 and GaborNet feature combination. This result was closely followed by the CL model trained on features from both ResNet18 and GaborNet, with a difference of 0.02. The GaborNet improved the result in these two cases.

Balance accuracy is another important evaluation metric used to measure the effectiveness of binary classifiers, especially when the classes are imbalanced. It is the average of the recall and specificity, as shown in Equation (17).

(17) $$Balance\;Accuracy=\frac{Recall+Specificity}{2}$$

CL with both ResNet18 and the GaborNet features achieved the highest balanced accuracy result, followed by CL with both ResNet50 and GaborNet features. The results indicate an improvement in the balance accuracy when the GaborNet features were fused with the ResNet features in both cases.

**Figure 6:**
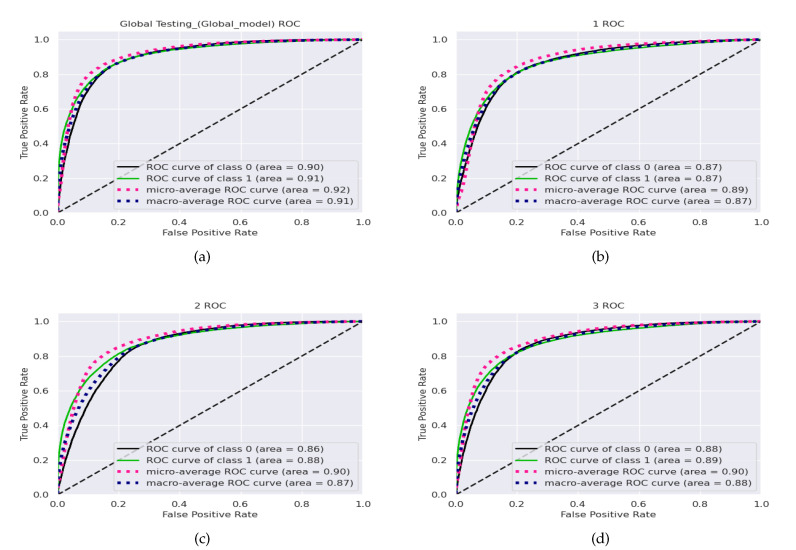
Receiver-operating curves for the (**a**) global model and the (**b**) client 1, (**c**) client 2, and (**d**) client 3 local models with the combined BHI test dataset and FL+ResNet50+GaborNet.

The receiver-operating curve (ROC) is a plot of recall against 1−specificity. Area under the curve (AUC) is the most widely used metric for measuring the overall performance of methods as it shows the chances that a randomly selected positive instance is more highly ranked than a randomly chosen negative one. Figure 6 shows the ROC curve and the corresponding area under the receiver-operating curve (AUC-ROC) results for each of the clients and the final global model for FL+ResNet50+GaborNet (the model with the best accuracy).

**Figure 7:**
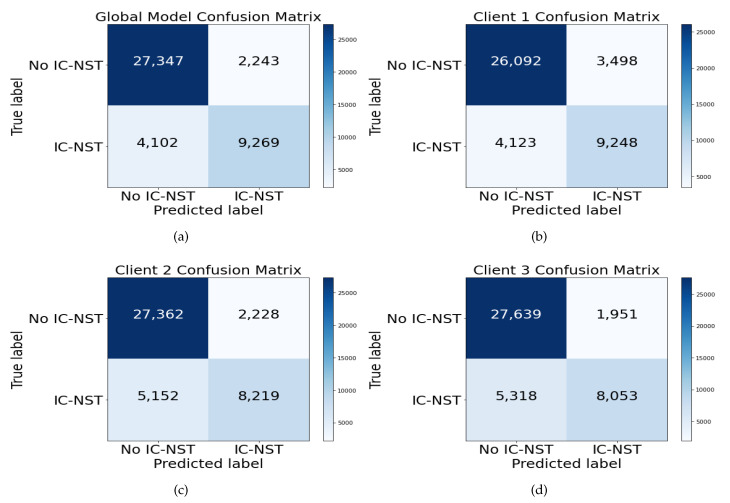
Confusion matrix for the (**a**) global model and the (**b**) client 1, (**c**) client 2, and (**d**) client 3 local models with the combined BHI test dataset and FL+ResNet50+GaborNet.

Confusion matrix helps visualize the number of actual and predicted instances of a dataset. Figure 7 shows the Confusion matrices of the FL+ResNet50+Gabor model on the BHI dataset for the global FL model, Figure 7a, and for the local models of each client, Figure 7b, Figure 7c, and Figure 7d. From the figures, all the local models performed well, given the imbalanced BHI dataset having more negative patch instances compared to the number of positive patch instances. The generalization of the local models were reflected in the performance of the global model.

### 4.3. Evaluation on the BreakHis Dataset

**Figure 8:**
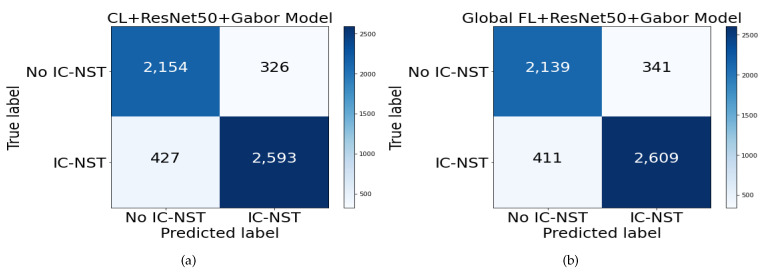
Confusion matrix for BreakHis dataset on (**a**) CL+ResNet50+Gabor and (**b**) FL+ResNet50+Gabor models.

To assess how well the trained models have generalized, we evaluated the performance of our approach on another domain data. The BreakHis dataset introduced in Section 3.4.2 was used to perform additional evaluations. All the 2480 images in both the subtypes and magnifying factor categories were combined for the benign class to form the negative (no IC-NST) instances. The malignant images were shuffled, and 3020 samples were randomly selected for the positive (IC-NST) instances. The results are reported in Table 4. From the table, the highest accuracy of 86.57% was obtained on the BHI dataset with our proposed FL+ResNet50+Gabor model. The corresponding accuracy with the same model for the BreakHis dataset is 86.32%, indicating only a 0.26% difference in the accuracy metric. However, the BreakHis dataset’s accuracy outperformed that of the BHI dataset on the CL+ResNet50+Gabor model. All the BHI results for specificity outperformed those of the BreakHis dataset, indicating that more negative targets were correctly detected from the BHI dataset. Conversely, all the recall results of the BreakHis dataset outperformed those of the BHI dataset, indicating the correct detection of more positive targets in the BreakHis dataset. These can be better visualized with the confusion matrices in Figure 8.

### 4.4. Comparison with Works in Literature

The BHI dataset is the most popular public IC-NST dataset; thus, we used it to compare with the results of other models in the literature. Table 5 shows the comparison in terms of accuracy, $F_{1}$ score, and the balance accuracy measure. From the table, our FL+ResNet50+GaborNet model outperformed the existing approaches in terms of accuracy and $F_{1}$ scores. The model proposed by Reza et al. [51] performed best in terms of balanced accuracy, with a value of 85.48%, surpassing our proposed ResNet18 + GaborNet with only a small margin of 0.29%. All our proposed models combining the ResNets with the GaborNet performed higher on the F1-score than the previous methods in the table.

## 5. Conclusions and Future Works

Invasive carcinoma of no special type is a deadly cancer that can be identified and classified by the microscopic similarity between cancer cells to normal tissue. Hence, a number of researchers have proposed methods using histopathological images to detect these kinds of cancers. Deep learning is one common technique for building such models; however, they require huge amounts of data, which is not always available in the medical field due to privacy concerns of patients.

In this research, we proposed an approach that leverages federated learning with pre-trained residual neural networks to securely train with local client data without sharing their training examples. We also introduced the Gabor network to extract features from the datasets and fused the feature map with the features extracted by the residual networks. A custom classifier network was used to obtain the predictions. We used popular evaluation metrics in the health domain to assess the performance of our approach on the BHI and BreakHis datasets. Analyzing the recall, specificity, and confusion matrices for both the BHI and BreakHis dataset indicated good generalization and the ability of the model to detect data imbalance and correctly classify the instances. Promising results were obtained without losing model quality, indicating the feasibility of federated learning for several possible applications, such as collaborative training in sensitive and privacy preservation scenarios, and interobserver variability assessment, among many others.

In future work, we intend to train each client on datasets obtained from different repositories, thereby introducing the limitations that come with such non-identically and independently distributed data sources. We will analyze it and propose approaches to limit the effects on the generalizability of the global model. We will also explore domain-based transfer learning techniques and recent advances for improving model performance on federated learning.

## Figures and Tables

**Table 1:** Private train and development splits for clients on the BHI dataset (patch-wise).

	Client 1	Client 2	Client 3
Train set	63,988	65,963	65,079
Dev set	11,966	13,252	12,561

**Table 2:** Patch-wise result comparison on the test datasets with the CL and the FL global models.

Metric	Precision (%)				Recall (%)				Specificity (%)			
	$C_{1}$	$C_{2}$	$C_{3}$	G	$C_{1}$	$C_{2}$	$C_{3}$	G	$C_{1}$	$C_{2}$	$C_{3}$	G
CL+ResNet18	-	-	-	78.46	-	-	-	77.37	-	-	-	90.40
FL+ResNet18	71.50	78.55	82.72	79.15	77.62	73.02	59.15	73.75	86.72	91.94	94.42	91.22
CL+ResNet18+Gabor	-	-	-	75.92	-	-	-	82.15	-	-	-	88.23
FL+ResNet18+Gabor	72.66	77.42	83.14	80.05	75.53	68.77	64.96	73.85	87.16	90.94	84.05	91.68
CL+ResNet50	-	-	-	79.18	-	-	-	75.13	-	-	-	91.07
FL+ResNet50	63.81	76.42	79.66	78.59	79.91	68.50	74.10	77.35	77.75	90.45	91.45	90.48
CL+ResNet50+Gabor	-	-	-	76.02	-	-	-	79.90	-	-	-	88.61
FL+ResNet50+Gabor	71.36	73.09	82.32	79.60	78.69	70.98	65.46	76.42	85.27	88.95	93.65	91.15

**Table 3:** Patch-wise comparison of our proposed models with studies from the literature on the BHI dataset.

Metric	Accuracy (%)				F1-Score (%)				BA Score (%)			
	$C_{1}$	$C_{2}$	$C_{3}$	G	$C_{1}$	$C_{2}$	$C_{3}$	G	$C_{1}$	$C_{2}$	$C_{3}$	G
CL+ResNet18	-	-	-	86.35	-	-	-	86.32	-	-	-	83.89
FL+ResNet18	83.00	83.66	83.44	85.78	83.09	83.01	82.57	85.64	81.82	78.64	76.78	82.48
CL+ResNet18+Gabor	-	-	-	86.33	-	-	-	86.47	-	-	-	85.19
FL+ResNet18+Gabor	83.54	84.04	84.99	86.13	83.62	83.76	84.43	85.97	81.34	79.85	79.50	82.77
CL+ResNet50	-	-	-	86.12	-	-	-	86.01	-	-	-	83.10
FL+ResNet50	80.57	84.18	86.05	86.40	81.20	83.36	85.91	86.36	82.28	80.92	82.78	83.92
CL+ResNet50+Gabor	-	-	-	85.90	-	-	-	86.00	-	-	-	84.26
FL+ResNet50+Gabor	84.02	82.84	84.88	86.57	84.28	84.47	84.35	86.49	83.27	79.59	79.55	83.78

**Table 4:** Patch-wise result comparison on the test datasets with the CL and the FL global models.

Datasets	BHI						BreakHis					
	Acc	Bac	F1	Pre	Rec	Spe	Acc	Bac	F1	Pre	Rec	Spe
CL+ResNet18	86.35	83.89	86.32	78.46	77.37	90.40	85.71	85.61	86.94	87.23	86.65	84.55
FL+ResNet18	85.78	82.48	85.64	79.15	73.75	91.22	84.14	84.15	85.34	86.63	84.11	84.19
CL+ResNet18+Gabor	86.33	85.19	86.47	75.92	82.15	88.23	85.47	85.42	87.00	87.40	85.93	85.00
FL+ResNet18+Gabor	86.13	82.77	85.97	80.05	73.85	91.68	86.02	86.01	87.12	88.14	86.13	85.89
CL+ResNet50	86.12	83.10	86.01	79.18	75.13	91.07	85.63	85.67	86.71	88.13	85.33	86.01
FL+ResNet50	86.40	83.92	86.36	78.59	77.35	90.48	84.91	84.90	86.08	87.22	84.97	84.84
CL+ResNet50+Gabor	85.90	84.26	86.00	76.02	79.90	88.61	86.31	86.36	87.32	89.00	85.86	86.85
FL+ResNet50+Gabor	86.57	83.78	86.49	79.60	76.42	91.15	86.32	86.31	87.40	88.44	86.39	86.52

**Table 5:** Patch-wise comparison of our proposed models with studies from the literature on the BHI dataset.

Paper	Model	Published Year	Accuracy (%)	F1 (%)	BA (%)
Cruz-Roa et al. [41]	CNN	2014		71.80	84.23
Janowczyk et al. [44]	AlexNet + Resize	2016		76.48	84.68
Reza et al. [51]	SMOTE	2018		85.78	85.48
Romano et al. [23]	CNN	2019		85.41	85.28
Kumar et al. [52]	CNN	2021	83.00		
Proposed (CL)	ResNet18+GaborNet	2022	86.33	86.47	85.19
Proposed (FL)	ResNet18+GaborNet	2022	86.13	85.97	82.77
Proposed (CL)	ResNet50+GaborNet	2022	85.90	86.00	84.26
Proposed (FL)	ResNet50+GaborNet	2022	86.57	86.49	79.55

## Data Availability

For our study, a publicly accessible dataset of breast histopathology images was used. A total of 277,524 patches were obtained from whole slide images (WSI) of subjects. 198,738 patches belong to the IC-NST negative class, whiles 78,786 patches belong to the IC-NST positive class [43,44]. A second dataset named Breast Cancer Histopathological (BreakHis) was used to perform ablation studies to validate the proposed models. The dataset consists of 2480 benign and 5429 malignant breast tumor tissue samples of different magnifying factors obtained from 82 patients. We used all the benign samples and 3020 malignant samples. The dataset is publicly available on the Laboratório Visão Robótica e Imagem website [45,46,47].

## Abbreviations

The following abbreviations are used in this manuscript:

IC-NST	Invasive carcinoma of no special type
IDC	Invasive ductal carcinoma
NOS	Breast cancer not otherwise specified
BRCA1/2	Breast cancer gene 1/2
CAD	Computer-aided diagnosis/detection
ML	Machine learning
DL	Deep learning
TL	Transfer learning
CL	Centralized learning
FL	Federated learning
$C_{1}$, $C_{2}$, $C_{3}$	Client 1, 2 and 3
CNN	Convolutional neural network
ResNet	Residual network
GaborNet	Gabor network
ReLu	Rectified linear unit
SGD	Stochastic gradient descent
LR	Learning rate
BHI	Breast histopathology image
BreakHis	Breast cancer histopathological
SOB	Surgical (open) biopsy
HE	Hematoxylin and eosin
Acc	Accuracy
Bac	Balance accuracy
F1	F1-score
Pre	Precision
Rec	Recall
Spe	Specificity
TP	True positives
TN	True negatives
FP	False positives
FN	False negatives
ROC	Receiver-operating curve
AUC-ROC	Area under the receiver-operating curve
